# Rapid Diagnostic Tests for Dengue Virus Infection in Febrile Cambodian Children: Diagnostic Accuracy and Incorporation into Diagnostic Algorithms

**DOI:** 10.1371/journal.pntd.0003424

**Published:** 2015-02-24

**Authors:** Michael J. Carter, Kate R. Emary, Catherine E. Moore, Christopher M. Parry, Soeng Sona, Hor Putchhat, Sin Reaksmey, Ngoun Chanpheaktra, Nicole Stoesser, Andrew D. M. Dobson, Nicholas P. J. Day, Varun Kumar, Stuart D. Blacksell

**Affiliations:** 1 Mahidol-Oxford Tropical Medicine Research Unit, Faculty of Tropical Medicine, Mahidol University, Bangkok, Thailand; 2 Centre for Tropical Medicine, Nuffield Department of Clinical Medicine, University of Oxford, Oxford, United Kingdom; 3 Institute of Child Health, University College London, London, United Kingdom; 4 Angkor Hospital for Children, Siem Reap, Kingdom of Cambodia; 5 Biological and Environmental Sciences, University of Stirling, Stirling, United Kingdom; University of Pittsburgh, UNITED STATES

## Abstract

**Background:**

Dengue virus (DENV) infection is prevalent across tropical regions and may cause severe disease. Early diagnosis may improve supportive care. We prospectively assessed the Standard Diagnostics (Korea) BIOLINE Dengue Duo DENV rapid diagnostic test (RDT) to NS1 antigen and anti-DENV IgM (NS1 and IgM) in children in Cambodia, with the aim of improving the diagnosis of DENV infection.

**Methodology and principal findings:**

We enrolled children admitted to hospital with non-localised febrile illnesses during the 5-month DENV transmission season. Clinical and laboratory variables, and DENV RDT results were recorded at admission. Children had blood culture and serological and molecular tests for common local pathogens, including reference laboratory DENV NS1 antigen and IgM assays. 337 children were admitted with non-localised febrile illness over 5 months. 71 (21%) had DENV infection (reference assay positive). Sensitivity was 58%, and specificity 85% for RDT NS1 and IgM combined. Conditional inference framework analysis showed the additional value of platelet and white cell counts for diagnosis of DENV infection. Variables associated with diagnosis of DENV infection were not associated with critical care admission (70 children, 21%) or mortality (19 children, 6%). Known causes of mortality were melioidosis (4), other sepsis (5), and malignancy (1). 22 (27%) children with a positive DENV RDT had a treatable other infection.

**Conclusions:**

The DENV RDT had low sensitivity for the diagnosis of DENV infection. The high co-prevalence of infections in our cohort indicates the need for a broad microbiological assessment of non-localised febrile illness in these children.

## Author Summary

DENV infection first manifests as an undifferentiated fever before either settling without complications, or progressing to severe disease requiring inpatient admission and careful supportive intravenous fluid management. The ability to differentiate DENV infection from other febrile illnesses, and to predict those at risk of severe disease is likely to be important. We assessed the diagnostic accuracy of a commercially available DENV rapid diagnostic test (RDT) for children admitted with febrile illness to a hospital in Cambodia during the DENV transmission season. We found sensitivity of the DENV RDT to be 58% and specificity to be 85% versus reference assay DENV serology. We then modelled the ability of clinical features, basic laboratory parameters, and DENV RDT result at presentation of the child to distinguish DENV infection from other febrile illness, and determine the need for critical care admission. We found that the DENV RDT did not increase the accuracy with which we diagnosed DENV infection, and was not helpful in deciding which children required critical care admission. Indeed, the relatively high prevalence of serious bacterial disease in the cohort of children indicated a broad microbiological differential diagnosis in all febrile children, regardless of their DENV infection status.

## Introduction

The number of people at risk of infection with one or more of the four dengue viruses (DENV) has increased exponentially over the past half-century, with the immuno-pathological complexity of DENV hampering vaccine design [1–4]. DENV infection first manifests as a non-specific febrile illness before either resolving, or progressing to severe disease characterized by endothelial activation, increased vascular permeability and impaired haemostasis [2,5]. Early diagnosis and identification of those at risk of severe disease is therefore important, leading to the development of rapid diagnostic tests (RDTs) to DENV antigens and anti-DENV antibodies [6–12], and previous work incorporating clinical and laboratory features of the disease (but not RDTs) into decision algorithms [13–18].

DENV infection is highly incident in Cambodia [19,20]. We examined our diagnostic strategy for DENV infection at Angkor Hospital for Children in Siem Reap, north-west Cambodia during the DENV transmission season of 2010. We used a DENV rapid diagnostic test (RDT) for detection of the DENV non-specific 1 (NS1) antigen, anti-DENV IgM, and anti-DENV IgG, [6–12,21] in addition to established clinical diagnostic criteria [22] and basic laboratory markers in children requiring hospitalization for a febrile illness with no clear source at admission. This study was nested within a prospective study of all causes of fever in hospitalized children [20], enabling us assess the DENV RDT in a well-characterised sample of children. In addition, we formalized our diagnostic algorithms with the use of conditional inference trees [13–18], and examined the usefulness of a DENV RDT for determination of risk of critical care admission in the context of highly prevalent co-infections.

## Methods

### Ethics statement

The parents of all children recruited to the study gave witnessed, written, informed consent before study enrolment. The Oxford Tropical Research Ethics Committee and Angkor Hospital for Children Institutional Review Board approved the study protocol on 24th September 2009 and 2nd October 2009 respectively.

### Clinical methods and enrollment

**Figure 1:**
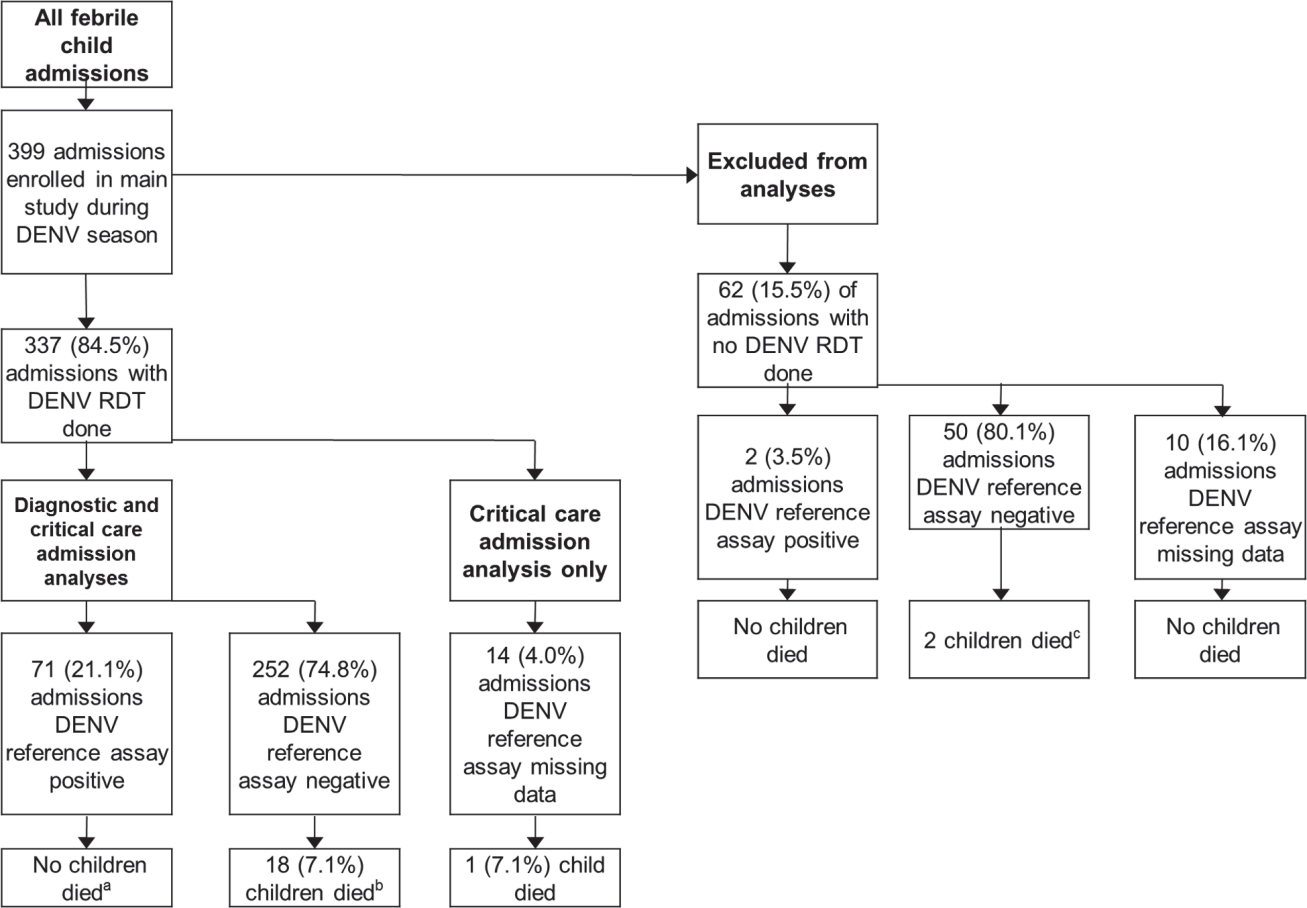
Flowchart depicting enrollment, DENV reference assay results, and outcome of patients into this study of a DENV RDT from the main prospective study of cause of fever in the cohort [20].

Criteria for enrollment in the parent study [20] were: age <16 years; fever ≥38°C within 48 hours of presentation to hospital; clinically requiring admission (using *Integrated Management of Childhood Illness* [*IMCI*] guidelines [23]); and written informed consent from the caretaker. Children <60 days of age were excluded (for reasons of blood volume). In addition, for this nested study, we prospectively excluded children with clear sources of fever on initial assessment: e.g. with clear lower respiratory tract infection, or gastroenteritis, or cellulitis (Fig. 1).

Children for this study were enrolled from 1st June 2010 to 1st October 2010. At enrollment, resident paediatricians examined all children. If a cause of fever was not clear, children were re-assessed by a senior paediatrician. Urinalysis was performed on all children. Admission findings were recorded on a study-specific clinical record form, which detailed clinical features associated with the diagnosis of dengue [22].

All children in this study had blood taken at enrollment for a DENV RDT, aerobic bacterial and *Leptospira* spp. culture, complete blood count, including malaria blood smear, nucleic amplification tests (NAATs) for rickettsial pathogens and *Leptospira* spp., and serum for biochemistry and DENV and Japanese encephalitis virus (JEV) serology. Where possible enrolled children also had a convalescent serum sample taken at 7 days or on hospital discharge for serology. Whole blood for NAATs and sera for serology were stored at -80°C and were analysed retrospectively on conclusion of the study. Children with a recent history of cough or sore throat and increased respiratory effort had nasal and throat swab samples analysed for respiratory virus detection. Samples of pus, cerebrospinal fluid (CSF), and other clinical tests were done as clinically indicated. Full methods are detailed in reference [20].

### RDT methods

We used the Standard Diagnostics (Korea) BIOLINE Dengue Duo [21] kit as the RDT in the study. This is an *in vitro* immunochromatographic, one-step assay for the detection of NS1 antigen and anti-DENV IgM and IgG from whole blood or serum, with manufacturer published sensitivity of 92.8% and specificity of 98.4% for NS1, and sensitivity of 99.4% and specificity of 93.0% for anti-DENV IgG and anti-DENV IgM [21]. NS1 antigenaemia is known to occur before IgM and/or IgG rise, thus assay of NS1 increases the sensitivity of DENV RDTs in early infection [6–12]. All testing was performed according to the manufacturer's instructions. In brief, 100μL of whole blood or serum sample was transferred by pipette into the sample well of the freshly unpackaged test device, and left for 20 minutes. A laboratory technician (one of five with experience in this RDT method), blinded to the clinical features of the child, interpreted the colour lines on the immunochromatographic strip. Indeterminate assay, or failed control, results were repeated once and counted as negative if the result was still indeterminate. Whole blood and serum intended for use with the DENV RDT was either used immediately following sampling, or refrigerated for a maximum of 48 hours at 5°C before being equilibrated to room temperature (approximately 25°C) prior to testing.

### Reference laboratory methods

The Panbio Japanese Encephalitis Dengue IgM Combo ELISA was retrospectively used for reference serology (Panbio, Australia; Cat. # E-JED01C; Lot # 110061) [24]. Panbio Units were calculated by multiplying the index value (calculated by dividing the sample absorbance by the cut-off value) by 10. The results were classed as negative for DENV and JEV if PanBio units were <9, indeterminate if 9–11 and positive if >11. If both anti-DENV and anti-JEV IgM results were positive, the anti-JEV result was divided by the anti-DENV result to give a ratio, with >1 indicating JEV infection and <1 indicating DENV infection (S1 Table). All ELISAs were repeated once if the positive, negative or calibrator samples were out of range, or the results indeterminate (repeated indeterminate results counted as negative). NS1 antigen ELISA (Standard Diagnostics, Korea; Lot # 224007) was used to detect DENV NS1 antigen [25].

To maximise specificity for the reference DENV assay we considered only children with a ≥4-fold rise in anti-DENV IgM titres or NS1 antigen positivity to be reference assay positive for acute DENV infection. We therefore excluded children who had a single high anti-DENV IgM titre (and no convalescent serum sample) (S2 Table).

### Statistical methods

All data was recorded onto a study-specific, password-encrypted database. Descriptive statistical analyses, summary statistics for the DENV RDT, and logistic regression analyses of clinical and laboratory data were undertaken on Stata v.12.1 (Stata Corp, TX, USA).

To capture the sequential availability of clinical/laboratory data available to an admitting paediatrician, we created three multivariate logistic regressions with reference assay positive DENV infection as the outcome variable. The first logistic regression included WHO clinical diagnostic criteria [22]: nausea and vomiting, rash, abdominal pain, mucosal bleeding (including rectal), reduced consciousness or confusion (infant/child Glasgow Coma Score ≤13 or disoriented in time or space), or palpable hepatomegaly, as independent binary variables. A second logistic regression included basic laboratory parameters (white cell count, platelet count, haematocrit, alanine transferase (ALT) level, C-reactive protein (CRP) level, malaria blood films), in addition to clinical features (above). The third logistic regression included clinical, laboratory, and DENV RDT variables (NS1 assay and IgM assay) to represent the addition of the DENV RDT to results to the decisive capabilities of the admitting paediatrician.

Three identical multivariate logistic regressions were undertaken (clinical data only; clinical data and basic laboratory parameters; and clinical data, basic laboratory parameters and DENV RDT results) but with critical care admission as the outcome variable.

Clinical, laboratory, and DENV RDT variables significantly associated (p<0.05) with either reference diagnosis of DENV infection or with admission to the critical care unit were entered into decision algorithms. We used recursive partitioning within a conditional inference framework, with statistical significance (permutation) testing for the generation of splits amongst independent variables (eliminating the need for a cross-validation step) using the R package *party* [26–28]. Similar analyses have been used previously [13–18] to develop diagnostic algorithms, although with variation in the statistical methods used.

We report the results using the Standards for the Report of Diagnostic accuracy studies (STARD)[29].

## Results

### Description of the cohort

399 febrile episodes in children ≥60 days of age were assessed between 1st June 2010 and 1st October 2010. Of these, 337 (84.5%) were tested with a DENV RDT. 62 (15.5%) children had clearly localised infections, were not tested with a DENV RDT, and are excluded from later analyses (Fig. 1, Table 1).

**Table 1:** Demographics, co-morbidities, clinical progress and final diagnosis for children with episodes of fever admitted to hospital during the dengue season 2010.

	RDT done n = 337	RDT not done n = 62	Significance
**Demographics**			
Male	178 (52.8%)	31 (50.0%)	p = 0.68^a^
Age (median, inter-quartile range [IQR])	3.4 (1.1, 8.1)	4.3 (1.7, 8.6)	p = 0.26^b^
**Comorbidities**			
Comorbid HIV infection	17 (5.0%)	1 (1.6%)	p = 0.23^a^
Mean z-score weight-for-age (95% confidence intervals [CI])^c^	–2.2 (–2.5, –2.0)	–2.2 (–2.5, –1.8)	p = 0.39^d^
Comorbid heart disease	17 (5.0%)	4 (6.5%)	p = 0.65^a^
**Clinical progress**			
Days of fever at presentation (95% CI)	4.4 (4.0, 4.8)	4.6 (3.5, 5.6)	p = 0.39^d^
Blood culture positive	17 (5.0%)	2 (3.2%)	p = 0.75^e^
Admission to critical care unit	70 (20.8%)	13 (21.0%)	p = 0.97^a^
Mechanical ventilation	26 (7.7%)	5 (8.1%)	p = 0.93^a^
Mortality	19 (5.6%)	2 (3.2%)	p = 0.42^a^ ^f^
**Final diagnosis**			
Reference ≥4-fold rise in anti-dengue IgM or NS1 antigen positive	71 (21.1%)^g^	2 (3.2%)^i^	p<0.0001^e^

^a^ Chi-squared test.

^b^ Mann-Whitney U test.

^c^ For those <5 years of age (n = 204 and n = 34, respectively).

^d^ Unpaired two sample t-test.

^e^ Fisher's exact test (2-sided).

^f^ 11 lost to follow-up.

^g^ 14 missing data and excluded (see Fig. 1).

^i^ 10 missing data.

Of children tested with a DENV RDT, 71/337 (21.1%) were reference assay positive for DENV infection (Table 1), 70 (20.8%) children were admitted to the critical care unit, and 19 (5.6%) children died. Comorbidities were prevalent: mean weight-for-age z-score was -2.2 (i.e. underweight, 95% confidence intervals [95% CI] –2.5, –2.0), known HIV infection was present in 17 (5.0%) children, and comorbid congenital or rheumatic heart disease in 17 children. The median number of days between acute and convalescent sera was 4 days (Interquartile Range [IQR] 3, 7).

### DENV RDT diagnostic accuracy evaluation

DENV RDT NS1 antigen alone had a sensitivity of 60.8% in comparison to reference NS1 assay, and RDT anti-DENV IgM had a sensitivity of 32.7% in comparison to reference anti-DENV IgM assay (Table 2). DENV RDT NS1 antigen was highly specific when compared to reference assay NS1 antigen (specificity 97.5%), but RDT anti-DENV IgM was less specific for reference assay anti-DENV IgM (specificity 86.2%). When combined, DENV RDT NS1 antigen and/or RDT anti-DENV IgM positivity had a sensitivity of 57.8%, specificity of 85.3%, positive predictive value of 52.6% and negative predictive value of 87.8% for reference assay positive DENV infection (Table 2).

**Table 2:** Sensitivity and specificity testing for a) DENV RDT NS1 versus reference assay NS1, b) RDT anti-DENV IgM versus reference assay anti-DENV IgM, and c) DENV RDT NS1 and/or RDT anti-DENV IgM versus reference assay positive DENV infection (see text for details of diagnostic criteria).

a)		DENV RDT NS1			
			Positive	Negative	Total
	**Reference NS1 assay**	Positive	31	20	51
		Negative	7	270	277
		Total	38	297	328
		Missing	2	7	9
	Sensitivity = 60.8% (95% CI 46.1, 74.2)				
	Specificity = 97.5% (95% CI 94.9, 99.0)				
**b)**		**DENV RDT IgM**			
			Positive	Negative	Total
	**Reference IgM assay (≥4-fold rise in titres)**	Positive	16	33	49
		Negative	38	237	275
		Total	54	270	324
		Missing	5	8	13
	Sensitivity = 32.7% (95% CI 20.0, 47.5)				
	Specificity = 86.2% (95% CI 81.5, 90.0)				
**c)**		**DENV RDT NS1 and/or IgM**			
	**Reference diagnosis of DENV infection**		Positive	Negative	Total
		Positive	41	30	71
		Negative	37	215	252
		Total	78	245	323
		Missing	4	10	14
	Sensitivity = 57.8% (95% CI 45.4, 69.4)				
	Specificity = 85.3% (95% CI 80.3, 89.5)				
	Positive predictive value = 52.6% (95% CI 40.9, 64.0)				
	Negative predictive value = 87.8% (95% CI 83.0, 91.6)				

Missing data was excluded from the sensitivity and specificity analysis.

### Covariates associated with DENV infection by reference assay or critical care admission

Table 3 shows adjusted Odds Ratios (aOR) and 95% CI for covariates associated with reference assay positive DENV infection, and critical care admission (for unadjusted variables see S3 Table).

**Table 3:** Adjusted odds ratios (aOR) for clinical features, laboratory parameters (including malaria blood films), and DENV RDT results, versus reference diagnosis of DENV infection, or all cause admission to the critical care unit (see text for details of regression analyses and adjusting covariates).

	Adjusted OR for reference diagnosis of DENV infection (95% CI)		Adjusted OR for all cause admission to critical care unit (95% CI)	
**Clinical features**		n		n
Nausea and vomiting^a^	**2.72 (1.42, 5.20)**	243	**0.37 (0.18, 0.77)**	255
Rash^a^	1.01 (0.35, 2.90)	243	0.42 (0.09, 1.97)	255
Abdominal pain^b^	**2.63 (1.37, 5.06)**	243	0.61 (0.27, 1.34)	255
Mucosal bleeding^b^	**0.23 (0.06, 0.80)**	243	1.01 (0.34, 3.02)	255
Reduced GCS or confusion^b^	0.39 (0.11, 1.42)	243	**7.04 (2.82, 17.57)**	255
Hepatomegaly^b^	1.21 (0.64, 2.31)	243	**2.98 (1.42, 6.26)**	255
**Laboratory parameters**				
Leukocyte levels (every 1.0 x10^9^/mm^3^ increase)	0.96 (0.89, 1.04)	191	**1.12 (1.04, 1.20)**	181
Platelet levels (every 10 x 10^9^/mm^3^ increase)	**0.95 (0.92, 0.98)**	191	1.00 (0.97, 1.02)	181
Haematocrit (every 1 percent increase)	0.99 (0.94, 1.06)	191	1.02 (0.96, 1.08)	181
Alanine transaminase (every 10 units/L increase)	1.00 (0.98, 1.02)	191	1.01 (0.98, 1.03)	181
C-reactive protein (every 1 mg/dL increase)	**0.76 (0.60, 0.95)**	191	**1.28 (1.05, 1.55)**	181
Blood films for malaria	0.22 (0.02, 2.04)	191	0.66 (0.07, 6.63)	181
**DENV RDT results**				
NS1 positive	**31.75 (6.79, 148.5)**	191	1.35 (0.25, 7.24)	181
NS1 and/or IgM positive	**4.19 (1.74, 10.11)**	191	0.64 (0.19, 2.12)	181
IgG positive and NS1 and/or IgM positive	**25.00 (6.15, 101.5)**	191	0.68 (0.15, 3.00)	181

^a^ Clinical signs of probable dengue [22];

^b^ clinical warning signs of dengue requiring inpatient management [22]

Bold type indicates p<0.05 for an association between covariate and outcome variable association. See S3 Table for unadjusted results.

At presentation, nausea and vomiting were associated with reference assay positive DENV infection (aOR 2.72, 95% CI 1.42, 5.20), as was abdominal pain (aOR 2.63, 95% CI 1.37, 5.06), whilst mucosal bleeding (including rectal) was negatively associated with reference assay positive DENV infection (aOR 0.23, 95% CI 0.06, 0.80). Children with mucosal bleeding were more likely to be diagnosed with gastroenteritis/dysentery as the cause of their febrile illness than those without mucosal bleeding: 21/59 (35.6%) versus 21/274 (7.6%) (χ^2^, p <0.001). Of laboratory parameters, platelet levels and CRP were negatively associated with reference assay positivity (aOR 0.97 per 10 x10^9^/mm^3^ increase, 95% CI 0.95, 0.99; and aOR 0.81 per 1 mg/dL increase, 95% CI 0.67, 0.99, respectively).

DENV RDT NS1 antigen positivity was strongly associated with reference assay positive DENV infection (aOR 31.75, 95% CI 6.79, 148.5), as was DENV RDT NS1 antigen and/or IgM (aOR 4.19, 95% CI 1.74, 10.11). Addition of DENV RDT IgG with NS1 antigen and/or IgM increased odds of reference diagnosis (aOR 25.00, 95% CI 6.15, 101.5).

70 (20.8%) children required admission to the critical care unit, 7 of whom were reference assay positive for DENV infection (no sub-analysis of these 7 children was possible). Amongst all causes, reduced consciousness and hepatomegaly were positively associated with admission to the critical care unit (aOR 7.04, 95% CI 2.82, 17.57; and aOR 2.98 95% CI 1.42, 6.26, respectively). Increased leukocyte level (aOR 1.12, 95% CI 1.04, 1.20 per 10 x10^9^/mm^3^ increase) and increased CRP level (aOR 1.28, 95% CI 1.05, 1.55 per 1 mg/dL increase) were also associated with critical care unit admission. Nausea and vomiting were negatively associated with admission to the critical care unit (aOR 0.37, 95% CI 0.18, 0.77). No other covariates, including the results of the DENV RDT, were associated with critical care unit admission. Children with missing observations within the variables regressed were excluded (Table 3).

### Decision algorithms with conditional inference trees

**Figure 2:**
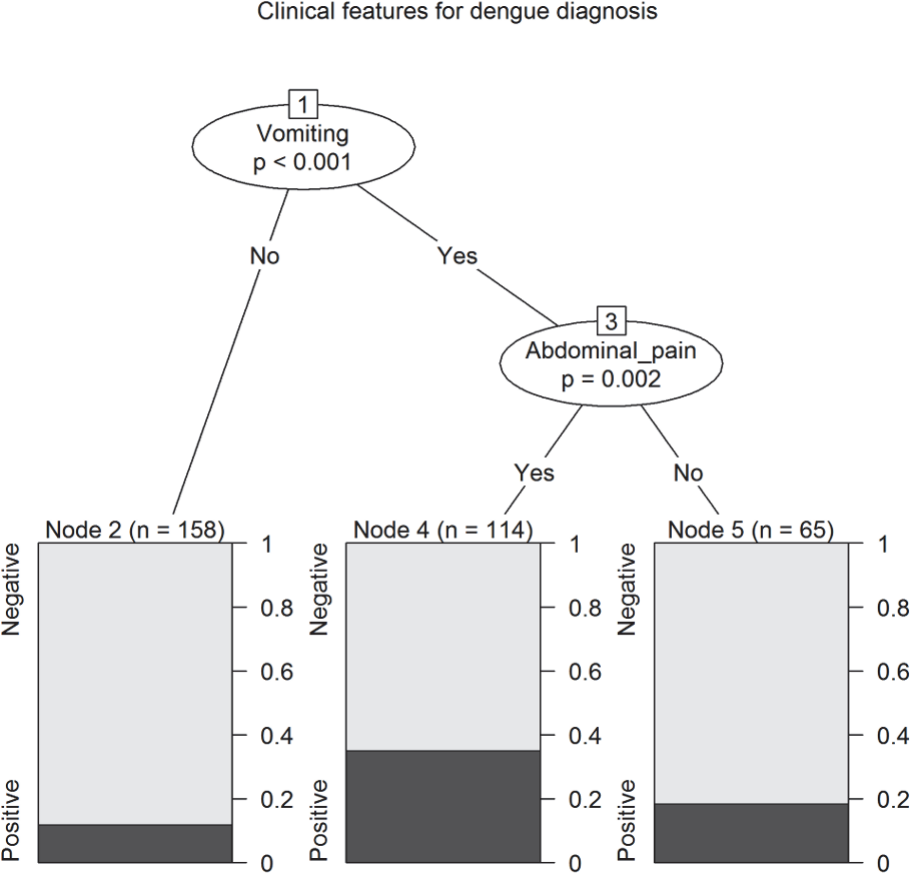
Conditional inference tree for the prediction of reference diagnosis of DENV infection using clinical diagnostics with an association (p<0.05) with reference diagnosis of DENV infection (nausea and vomiting and abdominal pain).

**Figure 3:**
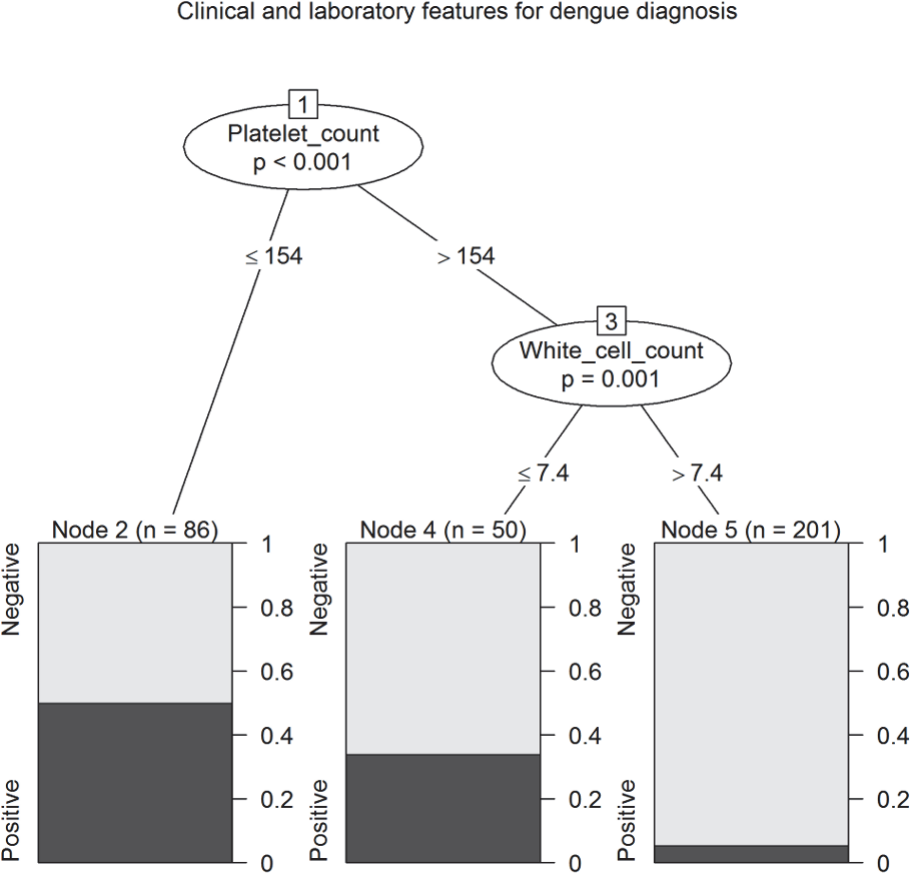
Conditional inference tree for the prediction of reference diagnosis of DENV infection using clinical features and laboratory parameters with an association (p<0.05) with the reference diagnosis of DENV infection (platelet levels and white cell levels; note the model removes clinical features from the tree).

From clinical covariates, nausea and vomiting and abdominal pain were selected as independent predictors of reference assay positive DENV infection (Fig. 2). When clinical and laboratory covariates were analysed together, children with a platelet level ≤154 x10^9^/mm^3^ were most likely to have reference assay positive DENV infection (although with an error rate of 50.0%) (Fig. 3). Children with a platelet level >154 x10^9^/mm^3^ and a leukocyte level >7.4 x10^9^/mm^3^ had a low likelihood of being reference assay positive for DENV infection (error rate 5.5%).

**Figure 4:**
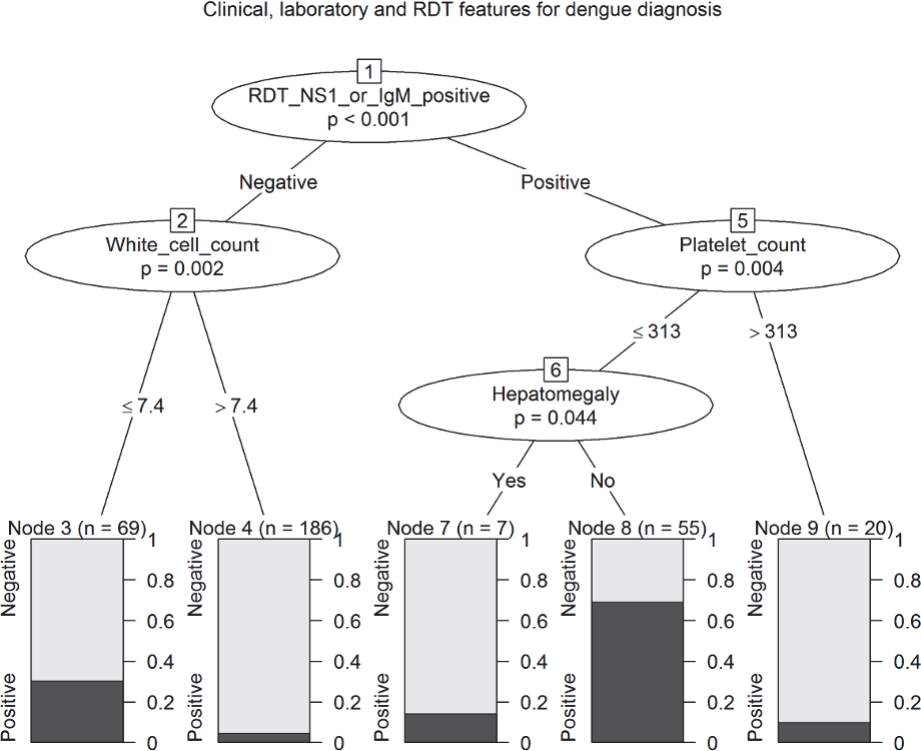
Conditional inference tree for the prediction of reference diagnosis of DENV infection using clinical diagnostics, laboratory parameters and DENV RDT results with an association (p<0.05) with reference diagnosis of DENV infection (DENV RDT, platelet levels, white cell levels, hepatomegaly).

Combining clinical, laboratory and DENV RDT covariates created five classifications (four, if node 6 [hepatomegaly] is combined) for likelihood of reference assay positive DENV infection (Fig. 4). Children with a positive DENV RDT result and platelet count ≤313 x10^9^/mm^3^ had the highest probability (error rate of 37.1%), particularly if they showed hepatomegaly (error rate 31.9%). Alternatively children with a negative DENV RDT, and white cell count >7.4 x10^9^/mm^3^ had a low likelihood (error rate 4.8%). Other classifications showed intermediate likelihood.

**Figure 5:**
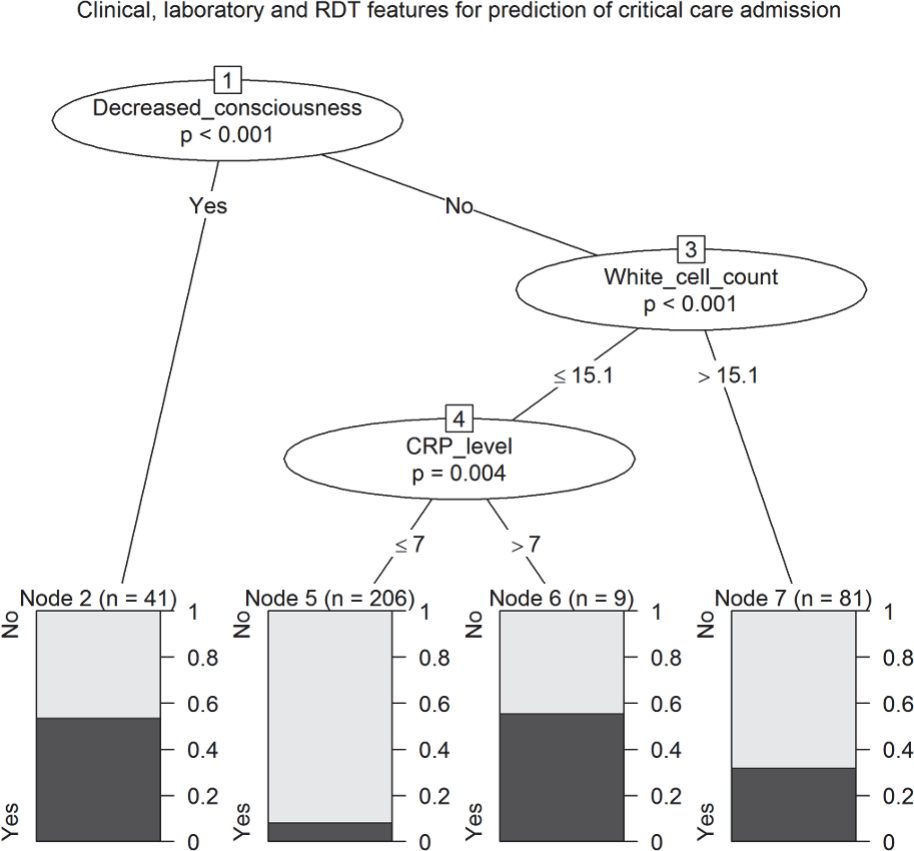
Conditional inference tree for the prediction of critical care unit admission using clinical diagnostics, laboratory parameters and DENV RDT results with an association with either the reference diagnosis of DENV infection, or all cause critical care admission.

Decreased consciousness was the most important classifier for all cause critical care admission (error rate 46.3%), whilst children with a white cell count ≤15.1 x10^9^/mm^3^ and CRP level <7 mg/dL were least likely to require critical care admission (error rate 8.3%) (Fig. 5).

### The problem of multiple diagnoses

Both DENV RDT positive and negative groups were infected with a range of other pathogens (Table 4). 22/82 (26.8%) children in whom a DENV RDT was positive had evidence of treatable infections: from serology, *Orientia tsutsugamushi* (eight children), *Rickettsia typhi* (five children); from direct stains, *Mycobacterium tuberculosis* (two children), *Plasmodium falciparum* (one child); from blood culture, *Burkholderia pseudomallei*, *Klebsiella pneumoniae*, *Salmonella enterica* serovar Typhi (all one child each); and from NAATs, *Leptospira* spp. (two children) and herpes simplex virus (one child, from CSF).

**Table 4:** Final microbiological (including virological/parasitological) diagnoses for patients, categorised by indication of acute DENV infection on DENV RDT result (NS1 and/or IgM positive; or NS1 and IgM negative).

	DENV RDT positive	DENV RDT negative
	n = 82	n = 255
**Flaviviruses**		
Dengue virus	41 (50.0%)	30 (11.8%)
Indeterminate flavivirus	1 (1.2%)	4 (1.6%)
Japanese encephalitis virus	5 (6.1%)	9 (3.5%)
**Rickettsioses**		
Orientia tsutsugamushi	8 (9.8%)	29 (11.4%)
Rickettsia typhi	5 (6.1%)	10 (3.9%)
Unknown *Rickettsia* spp.	0	3 (1.2%)
**Respiratory viruses**		
Respiratory syncytial virus	1 (1.2%)	4 (1.6%)
Influenza A H1N1-pdm09	0	4 (1.6%)
Parainfluenza virus 3	0	3 (1.2%)
Parainfluenza virus 1	0	1 (0.4%)
**Cultured bacteria**		
*Salmonella enterica* serovar Typhi	1 (1.2%)	8 (3.1%)
Burkholderia pseudomallei	1 (1.2%)	4 (1.6%)
Streptococcus pneumoniae	0	3 (1.2%)
Staphylococcus aureus	0	3 (1.2%)
Escherichia coli	0	2 (0.8%)
Haemophilus influenzae	0	1 (0.4%)
*Acinetobacter* spp.	0	1 (0.4%)
Klebsiella pneumoniae	1 (1.2%)	0
Bacteria of unknown significance	1 (1.2%)	2 (0.8%)
**Other**		
*Leptospira* spp	2 (2.4%)	2 (0.8%)
Mycobacterium tuberculosis	2 (2.4%)	1 (0.4%)
Plasmodium falciparum	1 (1.2%)	1 (0.4%)
*P*. *falciparum* and *Plasmodium vivax*	0	2 (0.8%)
P. vivax	0	1 (0.4%)
Enterovirus	0	1 (0.4%)
Herpes simplex virus	1 (1.2%)	0
Yeast (non-cryptococcal)	0	1 (0.4%)
Haematological malignancy	0	1 (0.4%)
Contaminant from blood culture	10 (12.1%)	18 (7.1%)
**Clinical diagnosis only**	8 (9.8%)	118 (46.3%)

## Discussion

During a single wet season in Cambodia a DENV RDT had a positive predictive value of 53% and negative predictive value of 88% for reference assay positive DENV infection in febrile children admitted to hospital. The high negative predictive value indicates that a negative DENV RDT may practicably "rule-out" DENV infection in this cohort (but *not* "rule-out" other co-infections).

As with other studies of diagnostic accuracy [6–18], this study is reliant on a gold standard test for its accuracy. The DENV RDT sensitivity of 58% was lower than similar studies [6–12], where sensitivity has ranged from 88% to 95% for identical or similar NS1/IgM combined proprietary DENV RDTs. Our study showed a lower incidence of reference assay positive DENV infection in comparison to these studies (possibly due to our deliberately broad inclusion criteria to encapsulate the breadth of DENV infection in infants and children [5], and high incidence of other infections), which may account for the apparent lower sensitivity. Alternative explanations for low sensitivity include differential sensitivity of the DENV RDT to circulating anti-DENV antibodies to different DENVs (DENVs 1–3 circulate in Cambodia [19]), or due to differing incidence of primary and secondary infections from other studies (both not assessed in this study). The exclusion of 14 children with only single acute serum samples (and NS1 antigen negative) did not adversely affect test sensitivity.

We found no evidence for a lower early IgM response in children in the cohort with younger age, or malnutrition, and no evidence that the variable length of time between acute and convalescent samples (and from onset of fever to presentation) reduce the sensitivity of the DENV RDT by producing false negatives by reference assay. Further studies would benefit from increased sample size, and sampling of convalescent sera after a fixed interval from the acute sample (although this may not be always feasible).

Incorporation of the DENV RDT result into conditional inference trees, with the use of platelet count (of ≤313 x10^9^/mm^3^), classified a high likelihood group (Fig. 4, nodes 7 and 8, error rate 37%) and a large low likelihood group (node 4, error rate 5%), with approximately a quarter of the cohort classified into one of two intermediate groups (nodes 3 and 9). However, a conditional inference tree generated by the same methods using platelet count (of ≤154 x10^9^/mm^3^) and leukocyte count (of ≤7.4 x10^9^/mm^3^) generated a more simple decision algorithm, with a high likelihood group (Fig. 3, node 2, error rate 50%), large low likelihood group (node 5, error rate 6%), and 15% in an intermediate group (node 4). Additionally, a positive DENV RDT result was not predictive of disease severity as judged by admission to the critical care unit; rather, critical care unit admission was predicted by reduced consciousness and *increased* leukocyte levels, and CRP ≥ 7 mg/dL (Fig. 5).

In brief, in this setting, the benefit of adding the DENV RDT to the diagnostic decision algorithm is therefore questionable, given presumed cost constraints. A complete blood count (with leukocyte and platelet levels), and CRP level were helpful for clinical decision-making regarding *both* diagnosis and disease severity, and should be undertaken on all children.

Was a positive DENV RDT indicative of the final diagnosis? No. Unlike many studies of DENV RDTs [6–12] and decision algorithms [13–18], our cohort of children had been thoroughly investigated in a study designed specifically to describe the microbiological causes of their febrile illness [20]. In this cohort nearly half of children with a positive DENV RDT result had a co-infection, over a quarter with a bacterial co-infection (Table 4).

The results of this study appear to contradict previous data on the utility of DENV RDTs. Retrospective analysis of the sensitivity and specificity of the RDT in known cases and controls, with a high "incidence" and clear-cut cases, would increase estimates of the diagnostic accuracy of the RDT [7–9]. Prospective studies, with higher DENV infection incidence (thus higher pre-test probability), have not integrated clinical and laboratory parameters into decision algorithms, increasing the apparent utility of DENV RDTs [6,11]. Amongst the elaboration of decision algorithms for DENV infection diagnosis, none have yet assessed the role of DENV RDTs [13–18].

Our emphasis on assessing DENV RDTs alongside other information attempts a realistic description of clinical decision-making. In future, improved DENV RDTs used in combination with RDTs to other important and locally prevalent pathogens may be helpful. In the meantime, good clinical observation, basic laboratory testing (including complete blood count, platelet levels and CRP), well-conceived "sepsis bundles" including antibiotic guidelines and resuscitation protocols, and sentinel microbiology facilities must continue [30,31].

## Supporting Information

S1 Table Classification of the Panbio Japanese Encephalitis Dengue IgM Combo and DENV NS1 antigen ELISA used for DENV reference assay: "Positive", "Negative" or "Missing" within the boxed region describes the final interpretation of the DENV reference assay (i.e. accounting both serology and NS1 antigen assay). Rise in titres is defined as ≥2 Panbio units between acute and discharge samples.
(DOC)

S2 Table Sensitivity and specificity testing for a) RDT anti-DENV IgM and b) DENV RDT NS1 and/or RDT anti-DENV IgM versus reference assay positive DENV infection (specified as reference NS1 antigen assay positive, or either ≥4-fold rise between acute and convalescent sera or, exceptionally in this Supplementary Material table, a single high titre of anti-DENV IgM on acute serology). Missing data was excluded from the sensitivity and specificity analysis.
(DOC)

S3 Table Unadjusted odds ratios (OR) for clinical features, malaria testing dengue RDT results and laboratory parameters, against DENV RDT NS1 antigen or RDT anti-DENV IgM positivity, or against reference assay positive DENV infection, or against critical care admission. OR in bold indicates p<0.05 for an association between covariate and outcome variable.
(DOC)

S4 Table a) Sensitivity and specificity of DENV RDT NS1, RDT anti-DENV IgM and RDT NS1 and/or anti-DENV IgM for confirmed reference diagnosis of DENV infection, by age of child. b) Sensitivity and specificity of DENV RDT NS1, RDT anti-DENV IgM and RDT NS1 and/or anti-DENV IgM for confirmed reference diagnosis of DENV infection, by the carer reported number of days of fever before presentation.
(DOC)

S1 Checklist STandards for the Reporting of Diagnostic accuracy studies (STARD) statement checklist. (DOC)
