# Correction: Rapid Diagnostic Tests for Dengue Virus Infection in Febrile Cambodian Children: Diagnostic Accuracy and Incorporation into Diagnostic Algorithms

**DOI:** 10.1371/journal.pntd.0003654

**Published:** 2015-03-19

**Authors:** The PLOS Neglected Tropical Diseases Staff

The species names in Table 4 are not correctly italicized. Please see the updated version of Table 4 for the correct italicization of species names.

**Table 4:** Final microbiological (including virological/parasitological) diagnoses for patients, categorised by indication of acute DENV infection on DENV RDT result (NS1 and/or IgM positive; or NS1 and IgM negative).

	DENV RDT positive	DENV RDT negative
	n = 82	n = 255
**Flaviviruses**		
Dengue virus	41 (50.0%)	30 (11.8%)
Indeterminate flavivirus	1 (1.2%)	4 (1.6%)
Japanese encephalitis virus	5 (6.1%)	9 (3.5%)
**Rickettsioses**		
*Orientia tsutsugamushi*	8 (9.8%)	29 (11.4%)
*Rickettsia typhi*	5 (6.1%)	10 (3.9%)
Unknown *Rickettsia* spp.	0	3 (1.2%)
**Respiratory viruses**		
Respiratory syncytial virus	1 (1.2%)	4 (1.6%)
Influenza A H1N1-pdm09	0	4 (1.6%)
Parainfluenza virus 3	0	3 (1.2%)
Parainfluenza virus 1	0	1 (0.4%)
**Cultured bacteria**		
*Salmonella enterica* serovar Typhi	1 (1.2%)	8 (3.1%)
*Burkholderia pseudomallei*	1 (1.2%)	4 (1.6%)
*Streptococcus pneumoniae*	0	3 (1.2%)
*Staphylococcus aureus*	0	3 (1.2%)
*Escherichia coli*	0	2 (0.8%)
*Haemophilus influenzae*	0	1 (0.4%)
*Acinetobacter* spp.	0	1 (0.4%)
*Klebsiella pneumoniae*	1 (1.2%)	0
Bacteria of unknown significance	1 (1.2%)	2 (0.8%)
**Other**		
*Leptospira* spp	2 (2.4%)	2 (0.8%)
*Mycobacterium tuberculosis*	2 (2.4%)	1 (0.4%)
*Plasmodium falciparum*	1 (1.2%)	1 (0.4%)
*P*. *falciparum* and *Plasmodium vivax*	0	2 (0.8%)
*P*. *vivax*	0	1 (0.4%)
Enterovirus	0	1 (0.4%)
Herpes simplex virus	1 (1.2%)	0
Yeast (non-cryptococcal)	0	1 (0.4%)
Haematological malignancy	0	1 (0.4%)
Contaminant from blood culture	10 (12.1%)	18 (7.1%)
**Clinical diagnosis only**	8 (9.8%)	118 (46.3%)
