# Correction: Rapid Diagnostic Tests for Dengue Virus Infection in Febrile Cambodian Children: Diagnostic Accuracy and Incorporation into Diagnostic Algorithms

**DOI:** 10.1371/journal.pntd.0004453

**Published:** 2016-02-05

**Authors:** Michael J. Carter, Kate R. Emary, Catherine E. Moore, Christopher M. Parry, Soeng Sona, Hor Putchhat, Sin Reaksmey, Ngoun Chanpheaktra, Nicole Stoesser, Andrew D. M. Dobson, Nicholas P. J. Day, Varun Kumar, Stuart D. Blacksell

The third author’s name is spelled incorrectly. The correct name is: Catrin E. Moore. The correct citation is: Carter MJ, Emary KR, Moore CE, Parry CM, Sona S, Putchhat H, et al. (2015) Rapid Diagnostic Tests for Dengue Virus Infection in Febrile Cambodian Children: Diagnostic Accuracy and Incorporation into Diagnostic Algorithms. PLoS Negl Trop Dis 9(2): e0003424. doi:10.1371/journal.pntd.0003424
