# Supplementary material for: Rapid Diagnostic Tests for Dengue Virus Infection in Febrile Cambodian Children: Diagnostic Accuracy and Incorporation into Diagnostic Algorithms
Source: PLoS Negl Trop Dis. 2015 Feb 24;9(2):e0003424. doi: 10.1371/journal.pntd.0003424 (PMC4340051; doi:10.1371/journal.pntd.0003424)
Supplement: S1 Table — Rise in titres is defined as ≥2 Panbio units between acute and discharge samples. (DOC) [file pntd.0003424.s001.doc]

**Table S1.**

|  |  | **NS1 antigen ELISA** |  |  |
| --- | --- | --- | --- | --- |
|  |  | Positive | Negative | Missing |
| **Panbio Japanese Encephalitis Dengue IgM Combo** | Rise in anti-DENV titres | *Positive* | *Positive* | *Positive* |
|  | Rise in anti-DENV titres > anti-JEV titres | *Positive* | *Positive* | *Positive* |
|  | Rise in anti-DENV titres < anti-JEV titres | *Positive* | *Negative^1^* | *Missing data^1^* |
|  | Static anti-DENV titres > anti-JEV titres | *Positive* | *Negative* | *Missing data* |
|  | Static anti-DENV titres < anti-JEV titres | *Positive* | *Negative* | *Missing data* |
|  | Fall in anti-DENV titres | *Positive* | *Negative* | *Missing data* |
|  | Low anti-DENV titres | *Positive* | *Negative* | *Missing data* |
|  | Single acute serum high anti-DENV titres | *Positive* | *Missing data^2^* | *Missing data* |
| ^1^ Counted as Indeterminate flavivirus in **Table 4**. ^2^ Counted as "missing data" in Main Article, but as "positive" in Supplementary Material **Table S2** (see text for details) | | | | |
