# Supplementary material for: Rapid Diagnostic Tests for Dengue Virus Infection in Febrile Cambodian Children: Diagnostic Accuracy and Incorporation into Diagnostic Algorithms
Source: PLoS Negl Trop Dis. 2015 Feb 24;9(2):e0003424. doi: 10.1371/journal.pntd.0003424 (PMC4340051; doi:10.1371/journal.pntd.0003424)
Supplement: S2 Table — Missing data was excluded from the sensitivity and specificity analysis. (DOC) [file pntd.0003424.s002.doc]

**Table S2.**

| **a)** |  | **DENV RDT IgM** | | | |
| --- | --- | --- | --- | --- | --- |
|  |  |  | Positive | Negative | Total |
|  | **Reference IgM assay (≥4-fold rise in titres or single high titre)** | Positive | 21 | 41 | 62 |
|  |  | Negative | 38 | 237 | 275 |
|  |  | Total | 59 | 278 | 337 |
|  |  | Missing | 0 | 0 | 0 |
|  | Sensitivity = 33.9% (95% CI 22.3, 47.0) | | | | |
|  | Specificity = 86.2% (95% CI 82.0, 90.0) | | | | |
| **b)** |  | **DENV RDT NS1 and/or IgM** | | | |
|  | **Reference diagnosis of DENV infection** |  | Positive | Negative | Total |
|  |  | Positive | 42 | 35 | 77 |
|  |  | Negative | 37 | 215 | 252 |
|  |  | Total | 79 | 250 | 329 |
|  |  | Missing | 3 | 5 | 8 |
|  | Sensitivity = 54.5% (95% CI 42.8, 65.9) | | | | |
|  | Specificity = 85.3% (95% CI 80.3, 89.5) | | | | |
