# Supplementary material for: Rapid Diagnostic Tests for Dengue Virus Infection in Febrile Cambodian Children: Diagnostic Accuracy and Incorporation into Diagnostic Algorithms
Source: PLoS Negl Trop Dis. 2015 Feb 24;9(2):e0003424. doi: 10.1371/journal.pntd.0003424 (PMC4340051; doi:10.1371/journal.pntd.0003424)
Supplement: S3 Table — OR in bold indicates p<0.05 for an association between covariate and outcome variable. (DOC) [file pntd.0003424.s003.doc]

**Table S3.**

|  | **Unadjusted OR for DENV RDT NS1 or RDT anti-DENV IgM positive (95% CI)** | | **Unadjusted OR for reference assay positive DENV infection (95% CI)** | | **Unadjusted OR for critical care admission (95% CI)** | |
| --- | --- | --- | --- | --- | --- | --- |
| **Clinical features** |  | **n** |  | **n** |  | **n** |
| Nausea and vomiting | **1.90 (1.14, 3.18)** | 335 | **3.16 (1.77, 5.65)** | 321 | 0.67 (0.39, 1.14) | 335 |
| Rash | 1.00 (0.45, 2.23) | 334 | 0.96 (0.40, 2.31) | 320 | 0.43 (0.15, 1.27) | 334 |
| Abdominal pain | **1.94 (1.12, 3.35)** | 261 | **3.11 (1.71, 5.63)** | 249 | **0.44 (0.23, 0.86)** | 261 |
| Mucosal bleeding | 0.57 (0.28, 1.19) | 333 | **0.42 (0.18, 0.98)** | 319 | 1.27 (0.67, 2.48) | 333 |
| Reduced GCS or confusion | 0.72 (0.32, 1.62) | 334 | 0.35 (0.12, 1.03) | 320 | **6.22 (3.12, 12.39)** | 334 |
| Hepatomegaly | 1.39 (0.84, 2.33) | 333 | 1.43 (0.84, 2.45) | 319 | **1.93 (1.12, 3.31)** | 333 |
| **Laboratory parameters** |  |  |  |  |  |  |
| Leukocyte levels (every 1.0 x10^9^/mm^3^ increase) | **0.95 (0.92, 0.99)** | 330 | **0.85 (0.80, 0.90)** | 316 | **1.07 (1.04, 1.11)** | 330 |
| Platelet levels (every 10 x 10^9^/mm^3^ increase) | **0.97 (0.95, 0.98)** | 329 | **0.95 (0.93, 0.97)** | 315 | 1.01 (0.99, 1.02) | 329 |
| Haematocrit (every 1 unit increase) | **1.07 (1.02, 1.11)** | 330 | **1.06 (1.01, 1.10)** | 316 | 0.98 (0.94, 1.02) | 330 |
| Alanine transaminase (every 10 units increase) | 1.01 (0.99, 1.03) | 323 | 1.00 (0.99, 1.02) | 310 | 1.01 (0.99,1.03) | 323 |
| C-reactive protein (every 1 mg/dL increase) | **0.76 (0.64, 0.90)** | 273 | **0.75 (0.63, 0.91)** | 261 | **1.22 (1.09, 1.37)** | 273 |
| Blood films for malaria | 1.27 (0.54, 3.00) | 337 | 0.44 (0.13, 1.51) | 323 | 1.30 (0.53, 3.20) | 337 |
| **Dengue RDT results** |  |  |  |  |  |  |
| NS1 positive | N/A |  | **27.13 (11.19, 65.77)** | 323 | 0.51 (0.19, 1.35) | 337 |
| IgM positive | N/A |  | **4.12 (2.23, 7.60)** | 323 | 0.55 (0.25, 1.21) | 337 |
| NS1 and/or IgM positive | N/A |  | **7.94 (4.42, 14.27)** | 323 | **0.45 (0.22, 0.93)** | 337 |
| NS1 and/or IgM positive, plus IgG positive | N/A |  | **12.69 (6.11, 26.39)** | 323 | 0.45 (0.17, 1.18) | 337 |
