# Supplementary material for: Rapid Diagnostic Tests for Dengue Virus Infection in Febrile Cambodian Children: Diagnostic Accuracy and Incorporation into Diagnostic Algorithms
Source: PLoS Negl Trop Dis. 2015 Feb 24;9(2):e0003424. doi: 10.1371/journal.pntd.0003424 (PMC4340051; doi:10.1371/journal.pntd.0003424)
Supplement: S4 Table — b) Sensitivity and specificity of DENV RDT NS1, RDT anti-DENV IgM and RDT NS1 and/or anti-DENV IgM for confirmed reference diagnosis of DENV infection, by the carer reported number of days of fever before presentation. (DOC) [file pntd.0003424.s004.doc]

**Table S4.**

| a) | **Age of child** | | |  |  |
| --- | --- | --- | --- | --- | --- |
| **Dengue RDT NS1** | **>60 days <1 year** | **≥1 year <5 years** | **≥5 years <16 years** |  |  |
| Sensitivity (95% CI) | 40.0% (12.2, 73.8) | 33.3% (14.6, 57.0) | 50.0% (33.8, 66.2) |  |  |
| Specificity (95% CI) | 96.9 (89.3, 99.6) | 99.0% (94.6, 100.0) | 95.4% (88.5, 98.7) |  |  |
| **RDT anti-dengue IgM** |  |  |  |  |  |
| Sensitivity (95% CI) | 50.0% (18.7, 81.3) | 28.6% (11.3, 52.2) | 37.5% (22.7, 54.2) |  |  |
| Specificity (95% CI) | 88.1% (77.8, 94.7) | 91.1% (83.8, 95.8) | 82.6% (72.9, 89.9) |  |  |
| **RDT NS1 and/or anti-dengue IgM** |  |  |  |  |  |
| Sensitivity (95% CI) | 50.0% (18.7, 81.3) | 47.6% (25.7, 70.2) | 65.0% (48.3, 79.4) |  |  |
| Specificity (95% CI) | 86.2% (75.3, 93.5) | 90.1% (82.5, 95.2) | 70.8% (60.7, 79.7) |  |  |
|  |  |  |  |  |  |
| b) | **Carer reported days of fever before presentation** | | |  |  |
| **Dengue RDT NS1** | **≤2 days** | **3 days** | **4 days** | **5 days** | **≥6 days** |
| Sensitivity (95% CI) | 44.4% (13.7, 78.8) | 26.7% (7.8, 55.1) | 50.0% (26.0, 74.0) | 64.7% (38.3, 85.8) | 23.1% (5.0, 53.8) |
| Specificity (95% CI) | 97.6% (91.5, 99.7) | 100% (91.4, 100) | 94.6% (81.8, 99.3) | 95.0% (83.1, 99.4) | 96.8% (88.8, 99.6) |
| **RDT anti-dengue IgM** |  |  |  |  |  |
| Sensitivity (95% CI) | 22.2% (2.8, 60.0) | 26.7% (7.8, 55.1) | 38.9% (17.3, 64.3) | 52.9% (27.8, 77.0) | 33.3% (9.9, 65.1) |
| Specificity (95% CI) | 92.7% (84.8, 97.3) | 92.7% (80.9, 98.5) | 86.7% (69.3, 96.3) | 67.6 (50.2, 82.0) | 90.3% (80.1, 96.4) |
| **RDT NS1 and/or anti-dengue IgM** |  |  |  |  |  |
| Sensitivity (95% CI) | 44.4% (13.7, 78.8) | 53.3% (26.6, 78.7) | 61.1% (35.8, 82.7) | 76.5% (50.1, 93.2) | 41.7% (15.2, 72.3) |
| Specificity (95% CI) | 90.2% (81.7, 95.7) | 92.7% (80.1, 98.5) | 83.3% (65.3, 94.4) | 64.9% (47.5, 79.8) | 87.1% (76.2, 94.3) |
